# Retirement Planning and Readiness of Local Government Employees in North‐Western Ghana

**DOI:** 10.1155/jare/5610286

**Published:** 2026-06-26

**Authors:** George Gyader, Ayeshetu Musah, Umar Haruna

**Affiliations:** ^1^ Department of History and Political Science, University of Business and Integrated Development Studies, Wa, Ghana; ^2^ Department of Governance and Development Management, University of Business and Integrated Development Studies, Wa, Ghana; ^3^ School of Public Health, Department of Social and Behavioral Change, University for Development Studies, Post Office Box, 1883, Tamale, Ghana, uds.edu.gh

**Keywords:** Ghana, local government employees, pension system, preparedness, retirement planning, Upper West Region

## Abstract

**Background/Objective:**

Retirement represents a significant life transition, yet limited research exists on retirement planning among public sector employees in sub‐Saharan Africa’s most impoverished regions. This study investigates the retirement preparedness of local government workers in Ghana’s Upper West Region, examining the factors that drive planning and readiness for retirement.

**Methods:**

A qualitative case study design was employed. Semistructured key informant interviews were conducted with 33 local government employees aged 25–45 years across five departments within all 11 metropolitan, municipal, and district assemblies of the Upper West Region. Participants were selected using purposive sampling with convenience recruitment. Data were analyzed using thematic analysis, guided by the life course perspective and theory of planned behavior.

**Findings:**

Key findings include (1) thoughts on retirement indicated that there was high awareness but age‐related postponement of planning; (2) retirement aspirations were guided by financial security, homeownership, healthcare access, and family time; (3) content of retirement plans included land acquisition prioritized over financial instruments due to eroded trust in banking institutions; (4) drivers of retirement planning included personal goals, family obligations, and notably, the absence of workplace retirement education programs; (5) overall preparedness showed inadequate preparation, complicated by cultural norms of wealth secrecy as a protective strategy against envy and social obligations.

**Conclusion:**

Local government employees in Northwestern Ghana demonstrate inadequate retirement preparedness. Effective interventions require context‐sensitive approaches addressing financial literacy, workplace education programs, and the cultural dynamics of savings disclosure in collectivist, resource‐constrained settings.

## 1. Introduction

Retirement represents a complex transition involving routine preparation activities alongside specific decisions linked to the timing and nature of retirement [1]. Although frequently perceived as an abrupt cessation of work activity, retirement is more accurately understood as a progressive transition shaped by financial, psychological, and social factors. The decision to retire is often difficult for employees due to uncertainties regarding finances, healthcare, and postretirement income [2]. Failure to understand retirement implications may lead to hasty decisions with long‐term negative consequences. Many individuals remain confused about retirement decisions and, due to general lack of awareness, are unable to plan properly. This lack of strategic consideration, combined with rising living costs, family burdens, and other social factors, adds considerable pressure on retirees.

Retirement planning is crucial in every worker’s life; however, it does not occur in isolation. Perceptions of and attitudes toward retirement vary considerably. While some view retirement as liberation from daily routines and an opportunity for leisure, others regard retirement as something to be feared and dreaded [3]. The thought of retirement in connection to economic well‐being typically creates anxiety among active workers [4]. This apprehension usually results from inadequate planning during employment. For some individuals, retirement means reduced working hours rather than complete exit from paid employment, while for others, it signifies complete withdrawal from the workforce [5].

The demographic landscape of the global population is undergoing significant transformation, characterized by increasing proportions of older age groups driven by rising life expectancy and declining birth rates [6]. Consequently, comprehensive research is needed to inform public policy and strategic planning regarding elderly populations’ needs and well‐being. Policymakers must prioritize studies that provide insights into challenges faced by this demographic and develop effective interventions supporting physical, mental, and social well‐being [7].

While existing literature on retirement planning has predominantly focused on developed economies and private sector employees, a notable gap exists in understanding retirement planning processes among local government workers in sub‐Saharan Africa, particularly in Ghana’s most impoverished regions. Specifically, no study has systematically examined: (1) how the unique socioeconomic conditions of Ghana’s Upper West Region characterized by a 70.7% poverty rate and predominantly rural population shape retirement planning behaviors among public sector workers; (2) the interplay between Ghana’s three‐tier pension system and individual‐level planning strategies; and (3) the cultural factors, including norms of communal support and secrecy around wealth accumulation, that influence retirement preparedness in collectivist societies. This study addresses these gaps by providing empirical evidence from local government employees in Northwestern Ghana.

## 2. Literature Review

### 2.1. Retirement Planning in African Contexts

Research on retirement planning in sub‐Saharan Africa remains limited compared to Western contexts. Studies from South Africa indicate that despite high awareness of retirement needs, actual planning behaviors lag significantly, particularly among middle‐aged employees [8]. In Nigeria, Maisango [4] found that preretirement anxiety among academic librarians was primarily driven by financial concerns, inadequate pension education, and perceived inadequacy of formal pension benefits. Across these contexts, common challenges include low financial literacy, limited access to formal savings instruments, high inflation eroding pension values, and weak social safety nets outside formal employment.

In Ghana specifically, Appiah‐Ansong [9] conducted a pioneering study on retirement planning programs (RPPs), finding that employees who attended such programs demonstrated more positive retirement attitudes and greater engagement in planning. However, coverage of such programs remains limited, particularly in rural and impoverished regions. Kumado and Gockel [10] highlighted that social protection in Ghana remains fragmented, with formal sector workers having significantly better retirement coverage than the informal majority.

### 2.2. Ghana’s Three‐Tier Pension System

The National Pensions Act (Act 766, 2008) established Ghana’s current three‐tier pension system, representing a significant reform of the previous SSNIT‐only framework. The three tiers comprise the following:

Tier 1 (Basic National Social Security Scheme): mandatory for all formal sector employees. Contributions are 13.5% of salary (5% employee, 8.5% employer), managed by SSNIT, providing a defined benefit at retirement. However, benefit adequacy has been questioned, with replacement rates typically low.

Tier 2 (Mandatory Occupational Pension Scheme): mandatory for formal sector employees, requiring 5% of salary (employee contribution) managed by private trustees licensed by the National Pensions Regulatory Authority (NPRA). This defined contribution scheme provides lump‐sum benefits at retirement.

Tier 3 (Voluntary Provident Fund and Personal Pensions): voluntary additional contributions beyond mandated tiers, allowing employees to supplement their retirement savings through approved fund managers.

Despite this comprehensive legislative framework, implementation challenges persist. These include delays in pension payments, limited financial literacy among employees regarding scheme options, erosion of benefits through inflation, and the exclusion of informal sector workers who constitute the majority of Ghana’s workforce. Notably, the Act’s provisions for employee education and counseling remain inadequately implemented, a gap this study explores empirically.

### 2.3. Determinants of Retirement Planning

International research has identified multiple determinants of retirement planning. Hershey et al. [11] found that many retirees failed to save during employment despite having financial capacity. Bonner [12] identified three primary reasons for inadequate retirement savings: insufficient lifetime earnings to save regularly, preference for immediate gratification over future savings, and failure to plan properly.

Riedel et al. [13] noted that retirement planning is a multifaceted, ongoing process requiring adjustment as circumstances evolve. Wang and Shultz [14] observed that retirement planning often does not occur until individuals approach actual retirement decisions, suggesting age as a critical moderator of planning behavior. Hennessey [15] emphasized that earlier retirement plan development and implementation increase the likelihood of meeting plan goals.

However, most of this research originates from Western contexts with well‐developed financial markets, strong regulatory frameworks, and different cultural norms regarding intergenerational support. The applicability of these findings to African contexts characterized by limited formal financial inclusion, extended family obligations, and cultural norms of communal resource sharing requires empirical investigation.

### 2.4. Homeownership and Retirement Readiness

Suh and James [16] found that homeownership serves not only as a social norm and milestone life event but also as a vehicle for wealth accumulation. Armstrong et al. [17] and Crawford [18] demonstrated that homeownership provides more options for funding retirement or reduces housing costs in retirement. In Ghanaian contexts, where mortgage markets are underdeveloped and rental housing is often insecure, homeownership assumes particular significance as a retirement strategy. However, rapid urbanization and increasing land prices make homeownership increasingly unattainable for many workers, particularly in regions like Upper West where poverty rates exceed 70%.

## 3. Theoretical Framework

This study is guided by two complementary theoretical frameworks: the life course perspective and the theory of planned behavior.

Life course perspective [19] posits that individual development and transitions must be understood within their historical, social, and cultural contexts. The perspective emphasizes several key principles relevant to retirement planning: (1) *timing*: the age at which individuals experience transitions shapes their outcomes; (2) *linked lives*: individuals are embedded in social relationships that influence their decisions; (3) *human agency*: individuals actively construct their life paths within structural constraints; and (4) *historical context*: cohort experiences are shaped by the period in which they live. Applied to this study, the Life Course Perspective helps explain how local government workers aged 25–45 years conceptualize retirement planning differently based on their career stage, how family obligations (linked lives) influence savings behavior, and how the specific historical context of Ghana’s banking crises and high poverty rates shapes attitudes toward formal retirement instruments.

Theory of planned behavior [20] provides a complementary framework for understanding the psychological mechanisms underlying retirement planning. The theory proposes that behavioral intention is predicted by three components: (1) attitudes toward the behavior (positive or negative evaluations); (2) subjective norms (perceived social pressure); and (3) perceived behavioral control (perceived ease or difficulty of performing the behavior). In this study, the Theory of Planned Behavior illuminates how workers’ attitudes toward retirement (whether viewed as opportunity or threat), subjective norms (workplace expectations, family pressures, community norms about savings disclosure), and perceived behavioral control (financial literacy, access to savings mechanisms, trust in financial institutions) collectively shape their retirement planning intentions and actual behaviors.

Together, these frameworks enable a comprehensive analysis that addresses both structural‐contextual factors (life course perspective) and individual psychological processes (theory of planned behavior), avoiding the limitations of a purely descriptive or purely individualistic approach.

## 4. Methodology

### 4.1. Study Area

The study was conducted in the Upper West Region of Ghana, one of 16 regions, carved from the former Upper Region in April 1983. The region comprises 11 municipalities and districts: Wa Municipal, Wa West, Wa East, Jirapa, Lambussie, Lawra, Nandom, Nadowli‐Kaleo, Daffiama‐Bussie‐Issah, Sissala East, and Sissala West. Situated in northwestern Ghana between longitudes 1°25″ West and 2°45″ East and latitudes 9°30″ N and 11°N, the region borders Burkina Faso to the north and west, the Savannah region to the south, and the Upper East and North East regions to the east.

The region spans 18,476 square kilometers, accounting for 12.7% of Ghana’s total land area. According to recent census data, the population is approximately 901,502 with a low density of 49 persons per square kilometer. Females constitute 52.1% of the population, while males account for 47.9%. The majority of residents (82.5%) live in rural areas. Age distribution shows 47% aged 0–14 years, 49% aged 15–60 years, and 4% aged over 60 years, resulting in a high dependency ratio.

Critically for this study, the region faces significant poverty challenges with a poverty rate of 70.7% the highest in Ghana. Severe food insecurity affects 63.6% of households. Despite having the highest National Health Insurance Scheme coverage at 81.9%, the health sector struggles with shortages of medical professionals.

### 4.2. Study Design

This study employed a qualitative case study design, particularly well‐suited for exploring complex social phenomena from multiple perspectives [21]. Qualitative approaches enable deeper insights by capturing nuances of human experience and highlighting the interplay of various factors. This design facilitates exploration of intricate psychological inquiries within specific cultural and social contexts [22]. The case study approach allowed researchers to delve into motivations and attitudes influencing retirement planning among local government employees by engaging participants in rich, contextual exploration of how the social environment, personal experiences, and cultural values shape retirement perspectives.

### 4.3. Study Sample and Sampling Procedure

Participants were purposively selected from staff members of all 11 Metropolitan, Municipal, and District Assemblies within the region. The local government service is structured into specialized units and departments. For this research, the sample was drawn from employees across five key departments within each assembly: Finance and Administration, Human Resources, Works and Sanitation, Community Development, and Agriculture. Inclusion criteria are as follows:

1. Permanent staff of the Local Government Service
2. Aged between 25 and 45 years
3. Employed in one of the five target departments
4. Willing to participate in the study

A purposive sampling approach was employed to select participants meeting specific inclusion criteria. Within each department, participants were recruited through convenience sampling based on availability and willingness to participate. This combined approach enabled access to information‐rich individuals while accommodating practical constraints of fieldwork in a resource‐limited setting.

Interviews were conducted iteratively, with data analysis proceeding concurrently with data collection. Thematic saturation, the point at which no new themes or insights emerged from subsequent interviews, was determined to have been reached after 30 interviews. Three additional interviews were conducted to confirm saturation, resulting in a final sample of 33 participants. This sample size is consistent with methodological literature indicating that saturation in homogeneous qualitative studies is frequently achieved between 12 and 30 interviews [23, 24].

### 4.4. Data Collection Procedure

A series of detailed key informant interviews were conducted to gather in‐depth data. The interviews were structured around a semi‐structured interview framework divided into three sections.

The first section focused on professional work profiles, exploring educational qualifications, career paths, specific organizational roles, and relevant experiences. The second section addressed the life transition of retirement. Through probing questions, respondents articulated their thoughts, feelings, and aspirations concerning retirement, including emotional responses, anticipated challenges, and envisioned postretirement activities. The third section concentrated on retirement preparation, examining behaviors, perceptions, and personal experiences regarding retirement planning, including financial readiness, lifestyle changes, and engagement in preparatory programs.

Interviews were conducted at times and locations convenient for respondents, creating a conducive environment for open dialog. Each session was recorded using electronic recording devices supplemented by manual note‐taking. Each interview lasted approximately 45 min.

Interviews were conducted in English by the lead researcher with assistance from two trained field assistants from the Local Government Service. The approach ensured smooth, respectful interactions throughout the interview process.

### 4.5. Data Processing and Analysis

All recorded interviews were transcribed. Electronic transcripts underwent rigorous cross‐referencing with manually written field notes to verify accuracy. After validation, a consolidated transcript sheet was created and cleaned for clarity and coherence.

Two researchers conducted an extensive review of all transcripts, engaging in careful reading and repeated revisiting of texts to identify prominent themes aligned with study objectives. Thematic analysis was conducted, involving coding data and categorizing it into relevant themes. Key quotes encapsulating main themes were selected, synthesized, and prepared for presentation.

The analysis was guided by the theoretical framework (Life Course Perspective and Theory of Planned Behavior), enabling interpretation of findings beyond mere description to address structural conditions and psychological mechanisms underlying retirement planning behaviors.

### 4.6. Ethical Considerations

Ethical approval was obtained from the University for Business and Integrated Development Studies (UBIDS) institutional review board. Informed consent was obtained from all participants. Anonymity and confidentiality were guaranteed, with pseudonyms used in reporting. Participants were informed of their right to withdraw at any time without consequences.

## 5. Findings

### 5.1. Sociodemographic Profile of Respondents

Twenty‐three respondents (69.7%) were male compared to 10 females (30.3%). Regarding religious affiliation, 60.6% subscribed to Islam and 39.4% to Christianity. Age distribution showed 30.3% aged 40–45 years, 27.3% aged 30–34, 27.3% aged 35%–39%, and 15.2% aged 25–29. The majority (78.8%) were married. Educational attainment was high, with 84.8% holding certificates, diplomas, or degrees, 12.1% holding master’s or doctoral degrees, and 3% having basic education only (see Table 1).

**TABLE 1:** Sociodemographic characteristics of respondents (*N* = 33).

Characteristic	Category	Frequency (*n*)	Percentage (%)
Gender	Male	23	69.7
	Female	10	30.3

Age group	25–29 years	5	15.2
	30–34 years	9	27.3
	35–39 years	9	27.3
	40–45 years	10	30.3

Marital status	Married	26	78.8
	Never married	5	15.2
	Separated/Divorced	1	3.0
	Widowed	1	3.0

Educational attainment	Basic education	1	3.0
	Certificate/Diploma/Degree	28	84.8
	Master’s/Doctoral degree	4	12.1

Religious affiliation	Islam	20	60.6
	Christianity	13	39.4
	Traditionalist	0	0.0

Department	Finance and Administration	8	24.2
	Human Resources	7	21.2
	Works and Sanitation	6	18.2
	Community Development	6	18.2
	Agriculture	6	18.2

*Note:* Source: Field Data, 2024.

### 5.2. Theme 1: Thoughts on Retirement

Respondents expressed varied thoughts regarding retirement plans and preparedness. Almost all respondents indicated they had, in one way or another, considered retirement. Age emerged as a determining factor: while younger employees were more likely to postpone retirement planning, middle‐aged employees demonstrated greater engagement with planning.

A 39‐year‐old respondent from the Finance and Administration Unit stated:

“Yes, I have thought of going on retirement. We are always reminded that we will not be here forever. I have worked with several people who are no longer with us, but they are still alive. They have gone home to take a deserved rest. No one can stay on forever, even if you wish to.”

A 43‐year‐old from the Community Development Department added:

“I have retirement plans. I was advised to put some money aside, get a side business, and get myself a comfortable home and means of transport. I was told to ensure that all the things my job entitles me to will be taken away when I retire, so I should prepare. That is why I am doing my best to make sure that by the time I get there, I will be able to go home fully prepared.”

However, some respondents, predominantly younger participants, admitted they had not seriously considered retirement. A 26‐year‐old from the Works and Sanitation Unit expressed:

“I am still too young to be considering retirement. I need to build my career first. I want to improve my qualifications and experience and slowly build my way to the top, then I can consider the matter of retirement. It is too far away and I am still new to the service. Retirement can wait.”

These findings confirm Wang and Shultz’s [14] assertion that retirement planning often does not occur until individuals approach actual retirement decisions. Through the Life Course Perspective, age‐based postponement reflects the principle of timing; younger workers perceive retirement as a distant life stage, reducing their sense of urgency for planning. The Theory of Planned Behavior further illuminates this finding: younger workers demonstrate positive attitudes toward retirement planning in principle but lack perceived behavioral control (financial capacity) while prioritizing immediate career development goals. This temporal discounting, valuing present over future rewards, represents a significant psychological barrier to early retirement planning in this population.

### 5.3. Theme 2: Retirement Aspirations

The main retirement aspirations articulated by respondents included financial security, comfortable postretirement living, and access to adequate healthcare. Additionally, owning a personal home emerged as a fundamental aspiration expressed by almost all respondents.

A 38‐year‐old from the Agriculture Department stated:

“I want to live a healthy and productive life after retirement. I have seen people retire and have nothing to show for it. They seem to have wasted their productive years and are now suffering. So I want to be financially secure and strong. I want to spend quality time with my grandchildren if God permits.”

Key informants consistently highlighted that spending quality time with family and close friends was a top priority, reflecting the importance of personal relationships. Financial security and access to adequate healthcare were identified as crucial aspects of plans.

Homeownership aspirations must be understood within Ghana’s socioeconomic context. In the absence of state‐provided retirement housing and underdeveloped mortgage markets, homeownership represents not merely a social norm or milestone event but a critical vehicle for wealth accumulation and postretirement security [16]. In the Upper West Region’s high‐poverty context (70.7% poverty rate), homeownership serves as a hedge against inflation, provides security for intergenerational transfers, and reduces postretirement housing costs [17, 18]. The emphasis on family time and healthcare access reflects the Life Course Perspective’s linked lives principle; retirement aspirations are not purely individualistic but embedded in family relationships and community structures. The Theory of Planned Behavior suggests that these strong positive attitudes toward retirement create favorable conditions for planning intentions, but perceived behavioral control (limited income after family obligations) remains a constraining factor.

### 5.4. Theme 3: Content of Retirement Plans

Land acquisition emerged as the most frequently cited retirement planning action, while retraining for another job, purchasing a second home, and real estate planning were either far from completion or not included in the overall plans.

A 42‐year‐old from the Community Development Unit explained:

“I think I am on course to fulfilling some targets for my retirement plans. I have acquired some parcels of land at strategic locations. I intend to use one of them to put up my retirement home, while the others remain investments.”

A 27‐year‐old from the Human Resource Unit added:

“Even though there is still a long way to go, I think acquiring land right away is the way to go. Land is fast disappearing and getting really expensive. So investing now sounds like a very good idea.”

Regarding preparedness across different areas (health, social networks, accommodation, leisure, financial stability), financial and accommodation concerns were most prominent, followed by health concerns. Leisure activities were considered less urgent, while social networks were regarded as least concerning.

A 37‐year‐old from the Finance and Administration Unit contributed:

“As for us, our culture guarantees that when I retire I will have a place among my peers in the social structure of my community hence I am not bothered about it. However, because poverty is high, I need to focus on saving a lot of money so that I will not be too dependent on others when I am on retirement. I will also need decent accommodation and access to healthcare. These are what I am planning for.”

The preference for land acquisition over formal financial instruments requires contextualization within Ghana’s recent banking sector crises. Multiple bank collapses and savings company failures (referenced explicitly by respondents in Theme 4) have severely eroded trust in formal financial institutions. Land, by contrast, represents a tangible asset perceived as immune to institutional failures a rational response to structural instability. This finding complicates Western retirement planning models that assume trust in formal financial markets. From the Life Course Perspective, respondents demonstrate human agency by adapting planning strategies to their specific historical and economic context. However, the emphasis on land over diversified financial instruments may concentrate risk, as land values are subject to market fluctuations, boundary disputes, and climate‐related pressures in an agricultural region.

### 5.5. Theme 4: Drivers of Retirement Planning

Personal goals and aspirations, financial resources, and family obligations played significant roles in shaping retirement plans. A 26‐year‐old from the Works and Sanitation Unit stated:

“How I plan my retirement depends on what obligations I currently have with my family. I am taking care of many people and so I have very little savings left to put aside for retirement. My personal goals are to develop myself and go as high as I can so that I will be able to retire comfortably.”

Regarding workplace retirement policies, respondents were asked whether they had received training or attended workshops on retirement. The results revealed that only a handful had ever attended preretirement education or counseling. A 35‐year‐old from the Finance and Administration Unit lamented:

“Unfortunately, even though it has always been touted that workshops and trainings form part of our routine human capacity development, I cannot recall the last time anyone organized one for us to discuss a topic on retirement and its preparation. I think this is very sad.”

A 29‐year‐old Human Resource officer confirmed:

“No, we have not been counselled or given any training on how to plan our retirement. There have been a few informal discussions on saving and investment opportunities by our union, but with the collapse of banks and savings companies, everyone is now afraid to even grant these people any audience. Ideally, the service should be proactive in giving staff training and information on pensions, savings and investment.”

Contrary to previous findings that workplace retirement policies like pension schemes and counseling workshops enhance knowledge and readiness [9], this study found that many local government workers were neither aware of nor permitted to participate in preretirement counseling. This represents a critical structural flaw within the local government establishment. The absence of workplace retirement education reinforces the Theory of Planned Behavior’s emphasis on perceived behavioral control; without knowledge and institutional support, employees perceive retirement planning as difficult, reducing their behavioral intentions. The reference to collapsed banks and savings companies reflects the historical context principle of the Life Course Perspective: the specific period in which these workers are planning (postbanking crises) shapes their distrust of formal financial institutions, driving preference for tangible assets. This finding extends Mushaphi’s [8] South African study, which found that despite most employees lacking retirement planning knowledge, some engaged in financial planning activities, suggesting that knowledge alone is insufficient; trust in financial systems is equally critical.

### 5.6. Theme 5: Overall Level of Preparedness

Comments from interviews indicated that overall preparation levels were not impressive. Some respondents felt there was still ample time to finalize plans, while others believed it was inappropriate to disclose planning activities. A 43‐year‐old from the Finance and Administration Unit offered a particularly revealing comment:

“My sister, if you want to live long in this our work and community, stop showing off or telling people what you are amassing. Don′t let anyone know what you are planning or what you have. It will only bring envy and that will not augur well for you. If you want to live up to retirement, pretend as if you have no plans for it. Even if you have saved a fortune towards it. Keep it to yourself.”

Many respondents conveyed complacency, believing there was still ample time to fine‐tune plans compared to older workers who might be closer to retirement.

This theme reveals a crucial cultural dimension absent from the Western retirement planning literature. The secrecy around retirement savings represents a rational protective strategy within a collectivist, high‐poverty context. Through the Life Course Perspective’s linked lives principle, individuals are embedded in social networks where wealth disclosure can trigger envy, requests for financial assistance, and social obligations that may deplete savings. The respondent’s use of “my sister” (a familial address term) underscores the blurred boundaries between workplace and kinship‐like social relations in Ghanaian contexts.

Research on social comparison theory suggests that individuals evaluate themselves relative to peers [25]. In team‐based environments, employees frequently value getting along with coworkers [26]. If coworkers perceive their own retirement as distant or unpredictable, sharing retirement plans indicating financial preparedness may cause workplace rifts. Chan et al. [27] found that retirement planning positively impacts psychological well‐being, but disclosing plans may be challenging to interpret accurately. Silver, Pang, and Williams [28] noted that candid conversations about retirement may impact workplace relationships, as close coworkers may believe the employee has already “checked out,” responding differently or even withdrawing.

This finding complicates conventional policy recommendations that assume that increased financial disclosure and planning transparency are universally beneficial. In the Upper West Region’s high‐poverty context, where 70.7% of the population lives below the poverty line, visible wealth accumulation may attract unwanted attention, accusations of corruption, or family obligations that deplete savings. The “silent savings” phenomenon represents an adaptive strategy requiring policy sensitivity rather than condemnation.

## 6. Discussion

This study examined retirement planning behavior among local government service workers in the Upper West Region of Ghana. The findings reveal a complex picture of awareness, aspiration, and constraint that extends existing literature by highlighting how structural conditions and cultural factors shape retirement planning in resource‐limited, collectivist contexts.

The finding that almost all respondents had thought about retirement indicates high awareness of retirement as a life transition. However, consistent with Wang and Shultz [14] and Hennessey [15], age significantly moderated planning behavior, with younger workers postponing active preparation. This temporal discounting represents a challenge for policy interventions, as the optimal window for retirement savings (early career, when compound returns are maximized) conflicts with the psychological tendency to defer planning until retirement approaches.

The strong preference for homeownership aligns with research demonstrating its importance for wealth accumulation and postretirement security [16, 17]. However, the emphasis on homeownership also reflects the inadequacy of formal pension provisions. In contexts where pension replacement rates are low and payment delays common, homeownership provides a tangible, controllable asset. Policy responses should consider whether promoting homeownership as a retirement strategy is appropriate or whether efforts should focus on strengthening formal pension systems.

The preference for land acquisition over formal savings reflects rational adaptation to Ghana’s recent banking crises. This finding contributes new empirical evidence to literature on retirement planning in contexts of financial sector instability, suggesting that trust in financial institutions is a necessary precondition for formal retirement savings. Without institutional trust, even employees with positive attitudes toward retirement planning will seek alternative, tangible assets even if those assets concentrate rather than diversify risk.

The finding that preretirement education and counseling are largely absent from local government workplaces contradicts Appiah‐Ansong’s [9] finding that RPPs positively influenced Ghanaian employees’ retirement attitudes. This suggests uneven implementation of retirement education programs across Ghana’s public sector, with the Upper West Region, Ghana’s poorest, being underserved. This geographic inequity in retirement education represents a policy concern requiring attention from the National Pensions Regulatory Authority and the Local Government Service.

The most distinctive finding, the culture of secrecy around retirement savings, extends the existing literature by highlighting how social dynamics in collectivist, high‐poverty contexts shape retirement planning disclosure. While Chan et al. [27] demonstrated that retirement planning benefits psychological well‐being, this study suggests that disclosing such planning may have negative social consequences. This finding has important implications for intervention design: programs that require public commitment or group disclosure of savings goals may be counterproductive in contexts where wealth secrecy is protective.

### 6.1. Implications for Ghana’s Three‐Tier Pension System

The findings raise questions about the effectiveness of Ghana’s Act 766 framework for workers in impoverished regions. While the three‐tier system provides comprehensive coverage on paper, implementation gaps, particularly absence of employee education, limit its practical impact. Moreover, the distrust of financial institutions revealed in this study suggests that Tier 2 (mandatory occupational pensions, privately managed) and Tier 3 (voluntary personal pensions) may face participation challenges without accompanying trust‐building interventions. The National Pensions Regulatory Authority should consider regionally differentiated education strategies that address specific barriers in high‐poverty contexts.

## 7. Conclusion

Employment plays a critical role in individuals’ lives, particularly for middle‐aged workers in the Local Government Service. This stage of life often brings heightened awareness of impending retirement transitions, generating various concerns among employees. This research found that local government employees’ attitudes toward retirement are generally positive, though notable negative sentiments cannot be overlooked.

A significant number of employees had contemplated retirement at various career points, reflecting common long‐term planning behavior. Optimistic views about retirement associated with freedom, leisure, and new opportunities evoked hope and anticipation. Conversely, negative perceptions contributed to anxiety about financial stability, loss of social interactions, and challenges of meaningful time use postretirement.

The most frequently considered proactive planning strategies included saving money and building retirement assets. Some respondents considered alternative options like purchasing second homes or extending employment duration. However, overall preparation levels remained inadequate, constrained by limited financial resources, family obligations, the absence of workplace education programs, and cultural norms discouraging savings disclosure.

Effective retirement planning is a vital component in determining postretirement quality of life. By addressing both positive aspirations and fears, local government employees can better prepare for futures aligned with their hopes while mitigating anxieties. However, individual‐level planning efforts require complementary structural supports: workplace retirement education programs, trustworthy financial institutions, pension payment reliability, and culturally sensitive interventions that respect norms around wealth secrecy.

### 7.1. Study Limitations

This study has several limitations. The qualitative case study design prioritizes depth over generalizability; findings may not transfer to private sector workers, informal sector employees, or other regions of Ghana. Self‐report data may be subject to social desirability bias, particularly regarding the sensitive topic of savings disclosure. The cross‐sectional design captures planning intentions and current behaviors but cannot track how plans evolve over time. Finally, the exclusion of retired workers means the study cannot compare preretirement intentions with postretirement outcomes.

### 7.2. Future Research Directions

Longitudinal studies tracking retirement planning from mid‐career through postretirement would throw more light on how plans translate into outcomes. Comparative studies between public and private sector workers, between different regions of Ghana, and between Ghana and other West African countries would identify context‐specific and transferable findings. Research explicitly examining the “silent savings” phenomenon, its prevalence, its relationship with actual savings behavior, and effective intervention strategies that respect secrecy norms is urgently needed. Finally, evaluation research assessing the impact of workplace retirement education programs (where they exist) would provide evidence for scaling effective interventions.

### 7.3. Policy Recommendations

Based on these findings, we recommend (1) mandatory preretirement education for all local government employees beginning at age 35, not only at retirement approach; (2) development of trust‐building communications regarding the safety of Tier 2 and Tier 3 pension instruments in the postbanking‐crisis context; (3) consideration of regionally differentiated pension education strategies addressing specific barriers in high‐poverty contexts like Upper West; (4) workplace savings programs that allow for private, confidential participation rather than group disclosure; and (5) strengthening of pension payment reliability to rebuild trust in formal systems.

## Funding

No funding was received for this manuscript.

## Conflicts of Interest

The authors declare no conflicts of interest.

## Data Availability

The data that support the findings of this study are available on request from the corresponding author. The data are not publicly available due to privacy or ethical restrictions.
